# Spatial links between subchondral bone architectural features and cartilage degeneration in osteoarthritic joints

**DOI:** 10.1038/s41598-022-10600-6

**Published:** 2022-04-23

**Authors:** Sara Ajami, Behzad Javaheri, Y.-M. Chang, Nimalan Maruthainar, Tahir Khan, James Donaldson, Andrew A. Pitsillides, Chaozong Liu

**Affiliations:** 1 Institute of Orthopaedics and Musculoskeletal Science, University College London, Royal National Orthopaedic Hospital, Stanmore, HA7 4LP UK; 2 Great Ormond Street Institute of Child Health, University College London, London, WC1N 1EH UK; 3 School of Mathematics, Computer Science and Engineering, City University of London, London, UK; 4 Comparative Biomedical Sciences, The Royal Veterinary College, Royal College Street, London, NW1 0TU UK; 5 Department of Orthopaedic Surgery, Royal Free Hospital, London, UK; 6 The Royal National Orthopaedic Hospital, Brockley Hill, Stanmore, HA7 4LP UK

**Keywords:** Bone, Cartilage, Osteoarthritis

## Abstract

Early diagnosis of osteoarthritis (OA), before the onset of irreversible changes is crucial for understanding the disease process and identifying potential disease-modifying treatments from the earliest stage. OA is a whole joint disease and affects both cartilage and the underlying subchondral bone. However, spatial relationships between cartilage lesion severity (CLS) and microstructural changes in subchondral plate and trabecular bone remain elusive. Herein, we collected femoral heads from hip arthroplasty for primary osteoarthritis (n = 7) and femoral neck fracture (n = 6; non-OA controls) cases. Samples were regionally assessed for cartilage lesions by visual inspection using Outerbridge classification and entire femoral heads were micro-CT scanned. Scans of each femoral head were divided into 4 quadrants followed by morphometric analysis of subchondral plate and trabecular bone in each quadrant. Principal component analysis (PCA), a data reduction method, was employed to assess differences between OA and non-OA samples, and spatial relationship between CLS and subchondral bone changes. Mapping of the trabecular bone microstructure in OA patients with low CLS revealed trabecular organisation resembling non-OA patients, whereas clear differences were identifiable in subchondral plate architecture. The OA-related changes in subchondral plate architecture were summarised in the first principle component (PC1) which correlated with CLS in all quadrants, whilst by comparison such associations in trabecular bone were most prominent in the higher weight-bearing regions of the femoral head. Greater articular cartilage deterioration in OA was regionally-linked with lower BV/TV, TMD and thickness, and greater BS/BV and porosity in the subchondral plate; and with thinner, less separated trabeculae with greater TMD and BS/BV in the trabecular bone. Our findings suggest that impairment of subchondral bone microstructure in early stage of OA is more readily discernible in the cortical plate and that morphological characterisation of the femoral head bone microstructure may allow for earlier OA diagnosis and monitoring of progression.

## Introduction

Osteoarthritis (OA) is the most prevalent joint disorder leading to a substantial socioeconomic burden^1^. OA is characterised by progressive loss of cartilage with concomitant structural and functional changes in the entire joint, including subchondral bone, ligaments, capsules and synovial membrane^2–4^. OA can impact any diarthrodial joint, including small hand joints and large knee and hip joints; the latter led to the UK second-highest number (95,677) of joint replacements in 2019^5^. OA symptoms often involve functional impairment and pain, and the predisposing risk factors include ageing, obesity, gender, trauma, genetics and mechanical loading^6,7^.

There are currently limited therapeutic options available to slow/reverse OA progression satisfactorily or provide effective and long-lasting symptomatic relief. This is partially due to the lack of a reliable method for early OA diagnosis, at which point intervention is likely to be more effective. The standard clinical feature for OA diagnosis is joint space narrowing reflecting articular damage/loss by radiography when clinical signs of pain and loss of mobility have already appeared^8,9^. However, at this stage, the joint is actively responding to the injury^10^. It has been reported that tissue-level alterations in subchondral bone occur before degeneration of the overlying cartilage^11,12^. Deciphering these changes may thus provide for earlier OA diagnosis and a greater understanding of aetiopathogenesis. Previous studies have attempted to describe these changes, yet the systematic correlation of these bone changes and their correlation to OA development in human joints remains to be elucidated.

High-resolution micro-computed tomography is sensitive enough to resolve spatial correlation between structural subchondral bone changes and macro/microscopic cartilage degeneration. Subchondral bone is classified as two anatomic phases: the subchondral plate and epiphyseal trabecular bone. Changes in subchondral bone microstructure in the femoral head have, however, mainly been assessed in its trabecular phase^13–18^. A recent study by Ryan et al.^19^ characterised the subchondral bone in its entirety and evaluated regional variations in bone morphometry within the femoral head. Analysing the entire femoral head may help to understand regional features of the bone microstructure and, in case of OA, would enable the additional resolution of site-specific structural changes and their spatial link to cartilage lesions of defined severity.

Previous studies in OA hips report thicker trabeculae, higher trabecular number and increased bone volume fraction at late OA compared to early-stage OA or normal hips^13,20–22^. Whilst these studies have significantly enhanced understanding of bone structural changes during OA development, the reported measures are based on defined segments/biopsies extracted from the femoral head^13,23–25^. To our knowledge the spatial relationship between cartilage lesion severity and structural changes in the local subchondral bone (plate/trabecular) across the entire femoral head is not fully defined. Herein, we explore whether quantifiable morphometric subchondral bone parameters that are highly related to cartilage lesion severity in human femoral heads can be identified. The additional data provided by our study, which analyses the entire femoral head can allow for a better understanding of the regional features of bone microstructure and site-specific structural changes at the same time as allowing generalised conclusions that are not impacted by any site-selection bias. Such an approach may lead to the development of predictive tools for early OA diagnosis in clinics. Moreover, investigating regional features of bone microstructure would provide more detail about the anatomy of the femoral head which would be useful for surgical interventions^26,27^.

## Materials and methods

### Sample collection

Following ethical approval (UK Health Research Authority REC reference: 15/LO/2052),13 human femoral heads were collected from patients undergoing hip arthroplasty for primary osteoarthritis (n = 7), and intracapsular fractures of the femoral neck (n = 6; non-OA controls). Informed consent was obtained from all participants and the research was performed in accordance with the Declaration of Helsinki.

For all patients, clinical records were reviewed and the following information was collected: gender, age at surgery, the reason for surgery and radiographic diagnosis (summarised in Table 1). Retrieved OA femoral heads were obtained from six females and one male patient with a mean age of 47.3 ± 17.7 years (range 33–72). The non-OA samples were retrieved from 6 females with a mean age of 79.2 ± 6.1 years (range 72–90). Samples were fixed in 10% neutral buffered formalin and stored in 70% ethanol at 4 °C until required.

**Table 1:** Relevant clinical data collected from patients at the time of surgery.

Patient number	Age (years)	Gender	Side	Reason for surgery	Radiographic diagnosis
P1	41	F	L	OA	Severe OA
P2	22	F	L	OA	Severe OA
P3	33	F	L	OA	Severe OA
P4	66	F	L	OA	Severe OA
P5	45	F	R	OA	Severe OA
P6	72	M	R	OA	Severe OA
P7	52	F	L	OA	Severe OA
P8	77	F	L	Trauma	Femoral neck fracture
P9	90	F	R	Trauma	Femoral neck fracture
P10	76	F	L	Trauma	Femoral neck fracture
P11	72	F	R	Trauma	Femoral neck fracture
P12	80	F	L	Trauma	Femoral neck fracture
P13	80	F	L	Trauma	Femoral neck fracture

### Cartilage lesion scoring by macroscopic assessment

**Figure 1:**
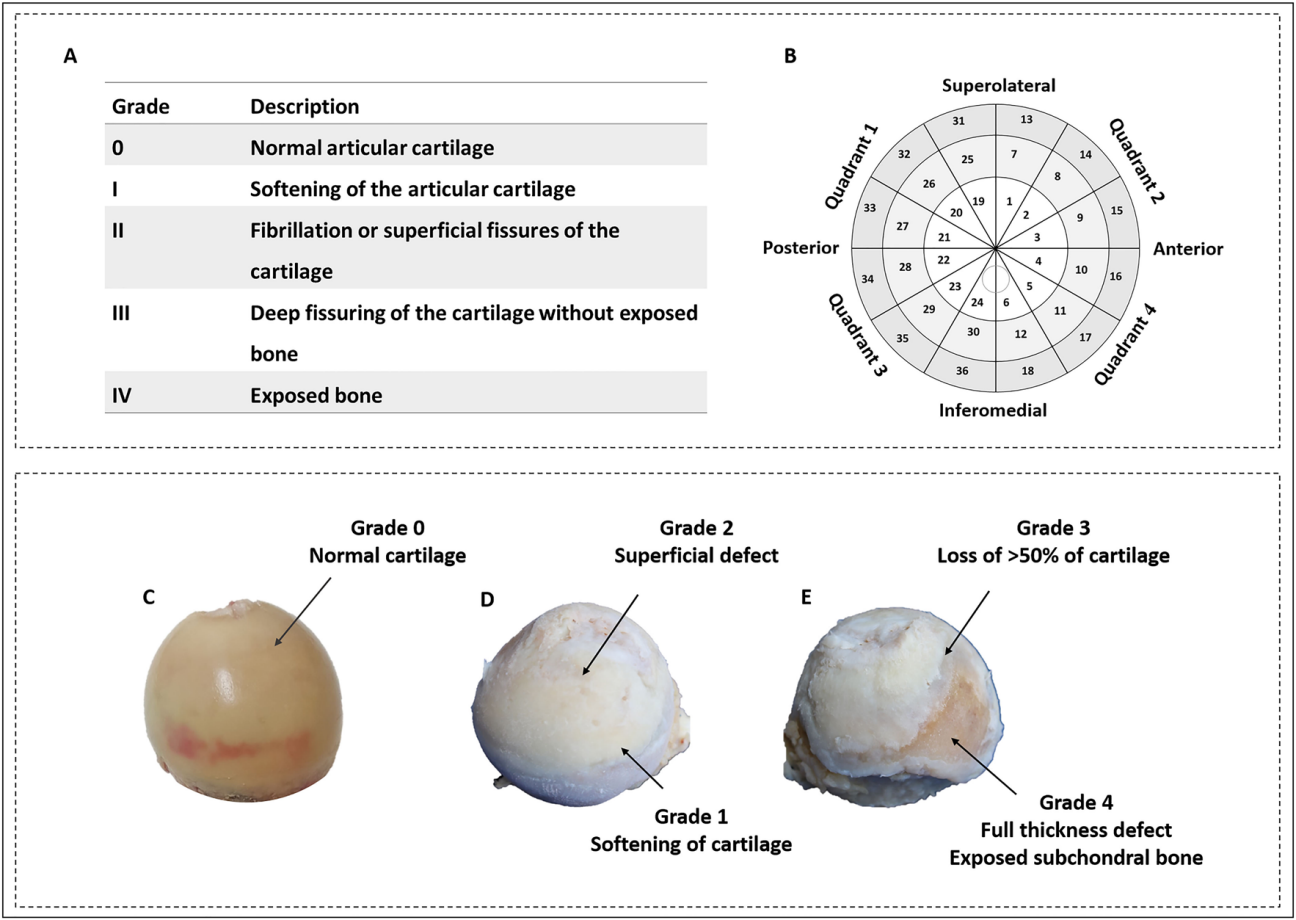
Severity mapping of osteoarthritic femoral heads. (**A**) Outerbridge classification used for grading cartilage lesions. (**B**) Schematic illustration of 36 regions for scoring cartilage lesion severity. (**C**–**E**) Femoral heads representing cartilage lesion severity of various grades. (**C**) Non-OA femoral head from a 77 year old female patient with normal articular cartilage. OA femoral head from a 52 year old female patient representing grades 1 and 2 (**D**), and 3 and 4 (**E**).

Cartilage lesion severity (CLS) was assessed using Outerbridge classification in all OA and non-OA samples by two independent observers (Fig. 1A) on a scale of 0–4^28^. Zero represents normal articular cartilage and 4 means full-thickness cartilage loss. Severity mapping of cartilage damage was used to visualise the anatomical distribution and severity of OA across the entire femoral head. Accordingly, each femoral head was divided into four quadrants: posterolateral (quadrant 1), anterolateral (quadrant 2), posteromedial (quadrant 3), and anteromedial (quadrant 4). Each region was further divided into three sub-regions, 30° apart. Radially, the femoral head was divided into three layers using methods described by Turmezei et al.^29^. Together, this provided 9 sectors in each quadrant and a total of 36 sectors in each femoral head (Fig. 1B). The macroscopic degeneration of cartilage (Fig. 1C–E) was then recorded in each sector and an average was obtained for each quadrant.

### High-resolution micro-computed tomography (micro-CT)

Each whole femoral head was scanned using a Skyscan 1172 (Skyscan, Kontich, Belgium), with X-ray tube operated at 80 kV and 124 μA, 2000 ms exposure time with a 0.5 mm aluminium filter and a voxel size of 13.46 μm. Scanning time was ~ 8 h for each sample. The slices were then reconstructed using NRecon 1.7.1.0 (Skyscan, Kontich, Belgium). 2D/3D analyses were performed using CTAn 1.17.7.1 + software (Skyscan, Kontich, Belgium). CTvox version 3.3.0 r1403 (Skyscan, Kontich, Belgium) was used for 3D visualisation. Calibrated micro-CT was used to assess cortical and trabecular tissue mineral density (TMD, defined as the mean density value of bone tissue voxels in a given region) using two Skyscan-supplied bone phantoms with known mineral density values of 0.25 and 0.75 g/cm^3^ calcium hydroxyapatite. The phantoms were scanned and reconstructed with the same scan settings.

### Morphometric analysis

Prior to analysis, micro-CT images were re-oriented in DataViewer 1.5.2.4 (Skyscan, Kontich, Belgium), such that fovea was medially located in trans-axial view. A dataset was then saved in trans-axial view. Using DataViewer, each femoral head was divided into four volumes of interest (VOI): Quadrant (Q) 1, Q2, Q3 and Q4 and degree of articular injury was recorded in each quadrant (Fig. 1B). Osteophytes were segmented manually from the bone structure in each quadrant using CTAn and binarized using a global minimum threshold value of 60–255 on 8-bit (0–255 Gray level) bitmap (BMP) images. The osteophyte volume (OV) and osteophyte volume fraction (OV/TV; %) were determined. Morphometric subchondral plate and trabecular bone parameters were evaluated separately, due to the possibility that these compartments may exhibit specific structural adaptations in OA, thereby creating a cortical (CORT) and trabecular (TRAB) segments in each VOI. A threshold of 57–255 was chosen to segment out the trabecular bone on 8-bit (0–255 Gray level) BMP images, and the structural parameters including bone volume fraction (BV/TV; %), trabecular thickness (Tb.Th; mm), number (Tb.N; mm^−1^), spacing (Tb.Sp; mm), bone pattern factor (Tb.Pf; mm^−1^), and bone surface (BS; mm^2^) were analysed using CTAn. For evaluation of the subchondral plate structure, the cross-sectional plate thickness (CS.Th; mm) and porosity (Po;%) were analysed in addition to BV/TV, BV, TV, BS and BS/BV.

### Statistical analysis

Statistical analyses were performed using SPSS (IBM SPSS Statistics 25.0 for Windows, IBM Corp, Chicago, IL, USA). All data were tested for normality using the Shapiro -Wilk test. A Mann Whitney-U test was used to compare medians for non-normally distributed numerical data. Values are reported as medians with interquartile range (IQR). The Spearman’s rank coefficient was used to assess whether there was a correlation between CLS and the structural parameters derived from trabecular, cortical and osteophyte analysis. CLS and age of patients were presented as mean ± standard deviation. In all cases, *p* values < 0.05 were considered significant.

Principal component analysis (PCA), a dimension reduction technique, was used to summarise measurements into a few uncorrelated principal components (PC) across quadrants. Structural parameters of trabecular bone and subchondral plate in the four anatomical regions were standardised (z-scores) and visualised using heatmaps. R Foundation for Statistical Computing, Vienna, Austria version 3.1.3 (http://www.r-project.org) was used for these analyses.

## Results

### Variation in trabecular and cortical plate architecture effectively distinguishes normal and OA femoral heads

Outerbridge scoring revealed CLS scores in OA patients ranging from 1.44 ± 0.94 to 3.22 ± 0.93 and, as expected, no signs of visible cartilage degradation in the non-OA control samples. Anatomically, CLS was most marked in quadrant 1, followed by quadrant 3, 4 and 2, although these differences did not reach levels of statistical significance (Table 2).

**Table 2:** Cartilage severity scoring according to outerbridge classification in the four anatomical regions defined; quadrant 1, quadrant 2, quadrant 3, quadrant 4.

Quadrant 1		Quadrant 2		Quadrant 3		Quadrant 4	
Patient1	1.11	Patient 1	1.00	Patient 1	1.89	Patient 1	1.78
Patient2	1.78	Patient 2	1.00	Patient 6	2.33	Patient 2	2.11
Patient3	2.33	Patient 6	1.89	Patient 4	2.44	Patient 6	2.11
Patient4	2.89	Patient 5	2.22	Patient 5	2.44	Patient 7	2.11
Patient5	3.11	Patient 7	2.33	Patient 7	2.56	Patient 5	2.22
Patient6	3.33	Patient 4	2.89	Patient 2	2.56	Patient 4	2.67
Patient7	3.44	Patient 3	3.33	Patient 3	2.78	Patient 3	3.00
Average	2.57		2.10		2.43		2.29

Evaluation of microstructural subchondral trabecular parameters in the seven OA and six non-OA femoral heads showed that surface area to bone volume ratio (BS/BV) was significantly higher in the OA group than non-OA control group (OA: 38.08 mm^−1^ [32.24–62.81] vs non-OA: 27.05 mm^−1^ [22.72–31.38], (*p* = 0.02)). This was coupled with thinner (OA: 0.10 mm [0.07–0.12] vs non-OA: 0.15 mm [0.13–0.17]) and less separated trabeculae in the OA samples (OA: 0.75 mm [0.65–0.82] vs non-OA: 0.99 mm [0.93–1.14], *p* = 0.01). Trabecular TMD was also significantly greater in OA compared to non-OA patients (OA: 0.37 g/cm^3^ [0.30–0.42] vs non-OA: 0.14 g/cm^3^ [0.10–0.22], *p* = 0.03). The strength of the link to CLS within the OA group was explored to show that samples with highest CLS (patients 5, 6 and 7) expressed significantly higher BS/BV, lowest Tb.Th and highest Tb.Sp; only exception was the sample from patient 2 in which highest Tb.Sp (1.09 mm [1.04–1.12) and lowest TMD (0.02 g/cm^3^ [0.02–0.4]) were evident. Moreover, significantly higher Tb.Pf indicated disrupted trabecular connectivity in OA samples. Patients 5, 6 and 7 had notably lower BV/TV, Tb.N and higher Tb.Pf when compared to control, non-OA samples.

Organisation of the subchondral cortical plate also differed, with significantly lower BV/TV (OA: 75.56% [73.88–78.59] vs non-OA: 85.76% [80.15–89.29], *p* = 0.2), higher BS/BV value (OA: 31.25 mm^−1^ [30.69–34.77] vs non-OA: 23.64 mm^−1^ [17.72–27.61], *p* = 0.01) and porosity (OA: 24.44% [21.41–26.12] vs non-OA: 14.24% [10.71–19.85], *p* = 0.02) in OA than in control non-OA samples. Trends towards lower TMD and cortical thickness were also observed in OA patients, but these differences were not significant.

**Figure 2:**
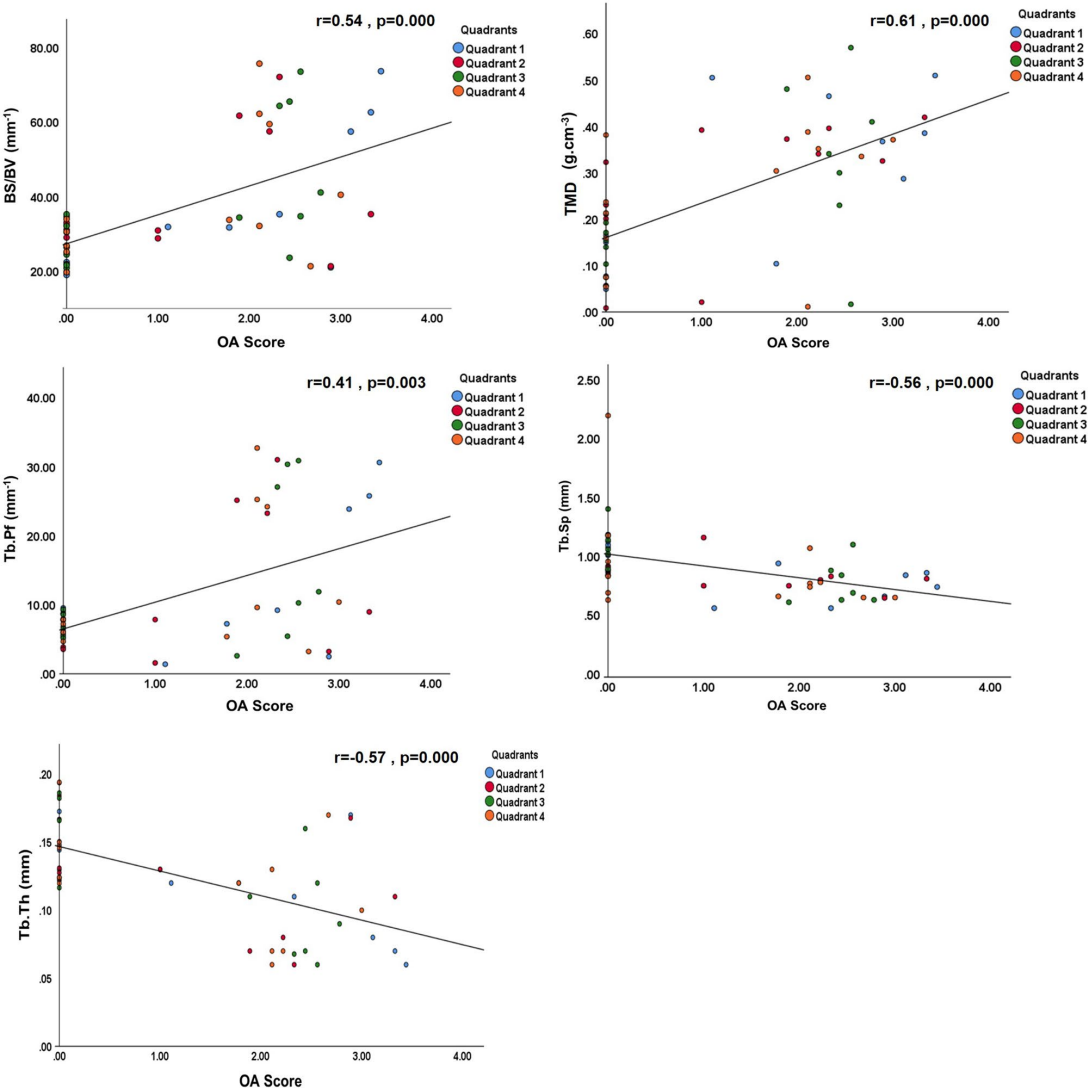
Trabecular bone correlation with OA severity in the entire femoral head. Positive correlation between OA severity and trabecular bone-specific surface (BS/BV), pattern factor (Tb.Pf), tissue mineral density (TMD). Negative correlation between OA severity and trabecular separation (Tb.Sp) and thickness (Tb.Th). *P* values and Spearman correlation coefficients are indicated on figures. *p* < 0.01 was considered significant.

Further interrogation of associations between cartilage degeneration and micro-CT parameters found a significant positive correlation in the trabecular bone between CLS score (Fig. 2) and entire femoral head BS/BV, Tb. Pf and TMD and, in contrast, significant negative correlation with Tb.Th and Tb Sp (*p* < 0.01). The validity of this approach was supported by the correlation between individual parameters which, as expected, showed higher BV/TV when trabeculae were thicker (r = 0.71, *p* < 0.0001) or more numerous (trabecular number, r = 0.85, *p* < 0.0001). Likewise, greater BS/BV was linked with lower Tb.Th (r = − 0.99, *p* < 0.0001) and higher Tb.Pf (r = 0.87, *p* < 0.0001). Negative correlations were also found between BV/TV and BS/BV (r = − 0.76, *p* < 0.0001), Tb.Sp (r = − 0.27, *p* = 0.04) and Tb.Pf (r = − 0.91, *p* < 0.0001).

**Figure 3:**
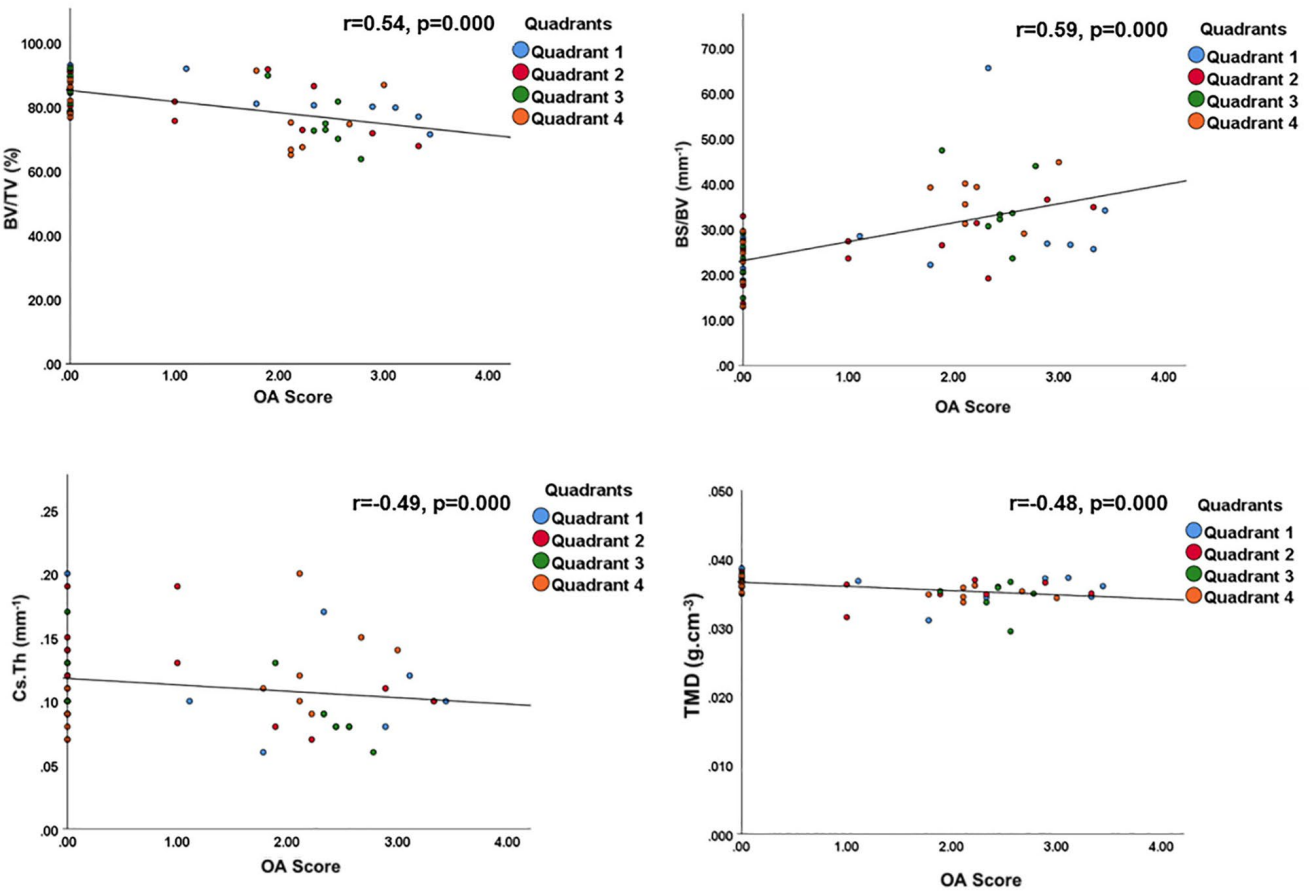
Subchondral plate correlation with OA severity in the entire femoral head. Bone volume fraction (BV/TV), thickness (Cs.Th), tissue mineral density (TMD), and bone-specific surface (BS/BV) presented as a function of OA severity. *P* values and Spearman correlation coefficients are indicated on figures. *p* < 0.0001 was considered significant.

Variation in cortical plate architecture also effectively distinguished OA femoral heads with differing CLS. Subchondral plate thickness, TMD and BV/TV were each significantly and negatively correlated with CLS, while BS/BV and porosity exhibited significant positive correlation (*p* < 0.0001, Fig. 3). As in trabecular bone, support for this approach was evident in the appropriate correlation between the measured parameters.

### Specific trabecular architectural parameters are related to OA cartilage lesion severity

**Figure 4:**
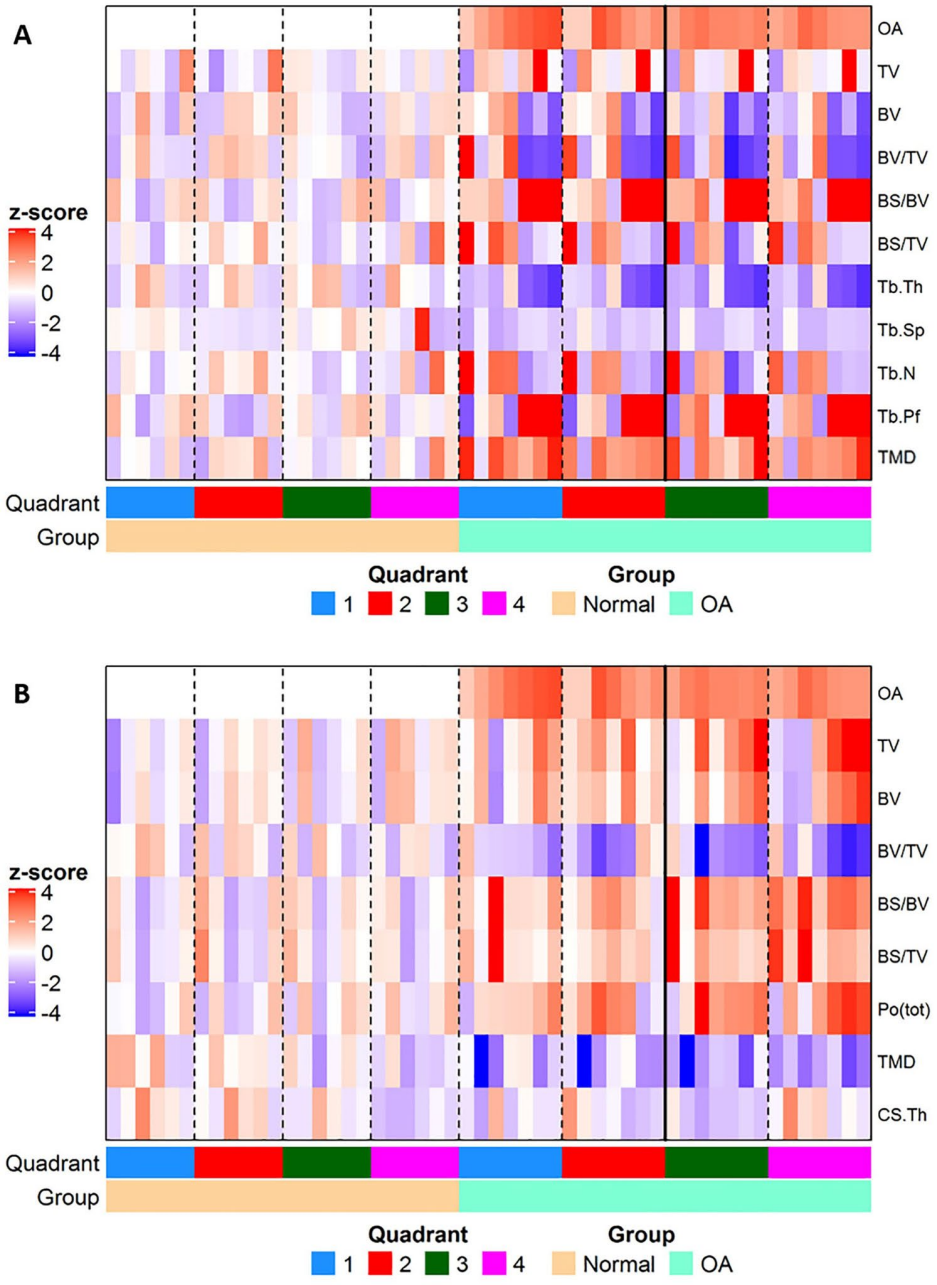
Heat maps showing differences in structural parameters of (**A**) trabecular bone and (**B**) subchondral plate in the four anatomical regions defined in both OA and non-OA samples. Patients were grouped according to disease status; all measurements were standardized (subtract mean and divide by standard deviation of non-OA controls) as indicated by z-scores (deeper red indicated higher readout and deeper blue indicated lower readout).

**Figure 5:**
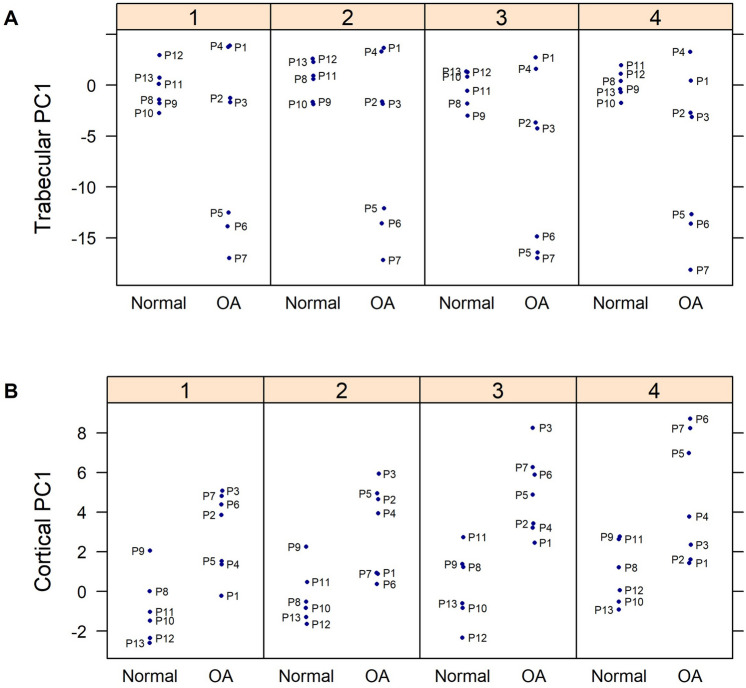
Figure showing principal component (PC 1) between OA and control samples at each quadrant for subchondral (**A**) trabecular and (**B**) cortical plate.

**Figure 6:**
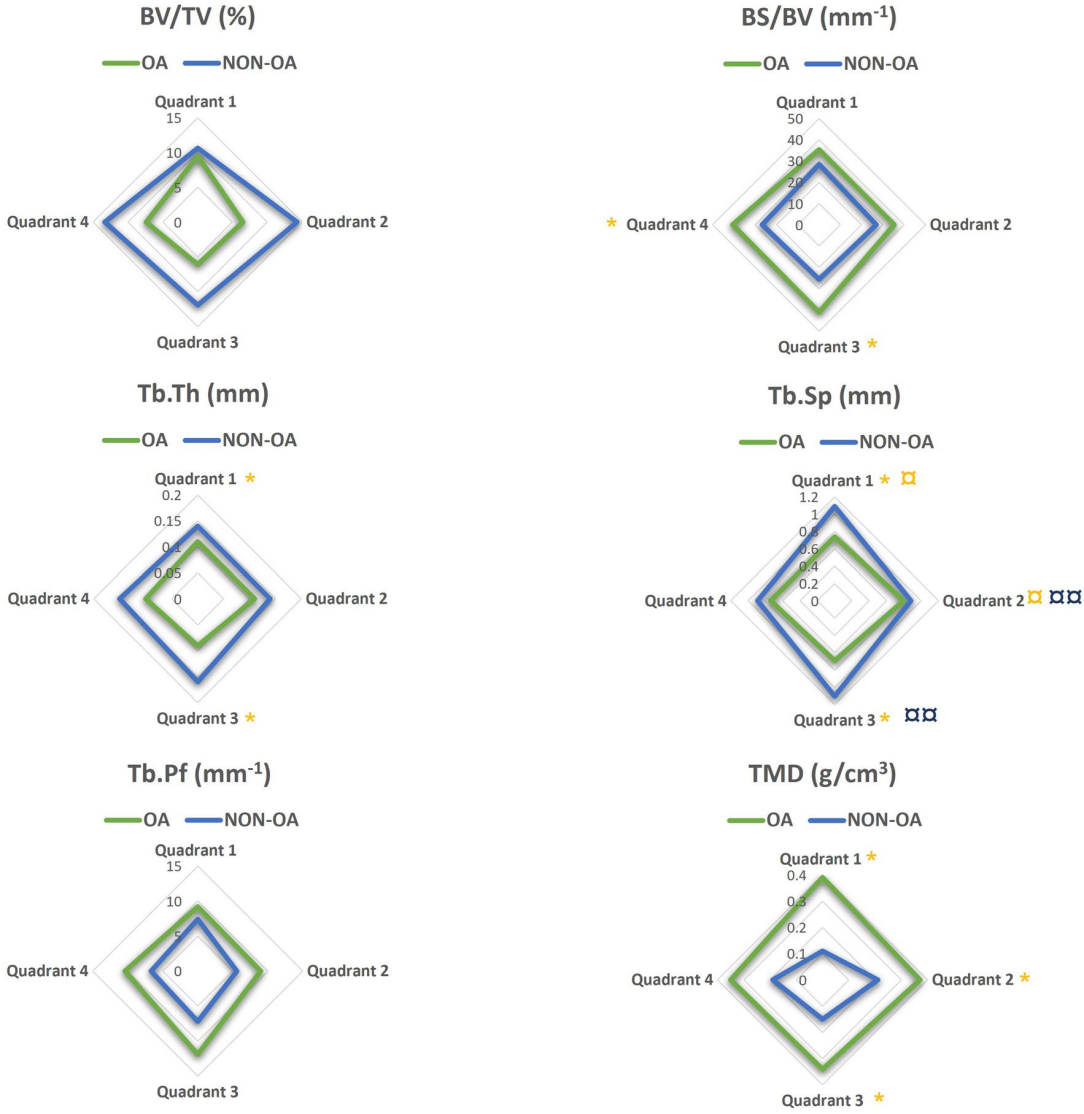
Regional distribution of the median value for the trabecular microstructural parameters evaluated in OA (green) and non-OA (blue) patients. No significant differences were found when subchondral trabecular in different anatomical regions were compared within the OA patients. (¤ and ¤¤) shows there is a significant difference between quadrants 1 and 2, and 2 and 3 respectively in non-OA patients. (*) shows a significant difference between OA and non-OA patients (*p* < 0.05). BV/TV: bone volume fraction; BS/BV: bone specific surface; Tb.Th: trabecular thickness, Tb.Sp: trabecular separation; Tb.Pf: trabecular pattern factor; TMD: tissue mineral density.

Mapping of microstructural changes in subchondral trabecular bone across all four femoral head quadrants and subsequent principal component analyses (PCA) indicated no significant structural difference in the trabecular compartment between OA and non-OA groups, as reported in Fig. 4. Intriguingly, detailed PCA investigation of the relationships within each quadrant showed a significant negative association between the first principle component (PC1) and CLS in both quadrants 1 and 3; OA patients 1–4 were similar to non-OA patients (Fig. 5A). For trabecular, the measurements contributed most to PC1 were Tb.Pf and BS/BV. PCA prompted further quadrant-by-quadrant interrogation (Fig. 6). This showed Tb.Th and Tb.Sp in quadrants 1 and 3 of OA samples were significantly lower than that of non-OA patients. Changes in TMD levels were more extensive, and were significantly higher in quadrants 1, 2 and 3 in OA samples than non-OA (Supplementary Table S1). OA-related BS/BV changes showed some divergence in distribution, with significant increases evident in quadrants 3 and also quadrant 4 in OA patients.

These quadrant-specific changes in trabecular architecture in the OA patients led us to examine whether they might be linked spatially with CLS. It was observed that a significant positive correlation in quadrants 1 and 3 between CLS and BS/BV, and a negative correlation with Tb.Th. CLS was also positively correlated with TMD in quadrants 1 and 3, whilst Tb.Sp was linked negatively to CLS in quadrants 1, 2, and 3 (Table 3).

**Table 3:** Correlation between OA severity and microstructural parameters in all quadrants in trabecular bone.

Quadrants	BS/BV		Tb.Th		Tb.Sp			TMD	
	1	3	1	3	1	2	3	1	3
r	0.63	0.62	− 0.76	− 0.59	− 0.71	− 0.67	− 0.72	0.75	0.62
*p*	0.02	0.02	0.002	0.03	0.01	0.01	0.01	0.003	0.02

BS/BV, bone specific surface; Tb.Th, trabecular thickness, Tb.Sp, trabecular separation; TMD, tissue mineral density. The correlation coefficient, r, and *p* values are reported. *p* < 0.01 was considered significant.

### Specific subchondral bone plate architectural imaging biomarkers are related to OA cartilage lesion severity

**Figure 7:**
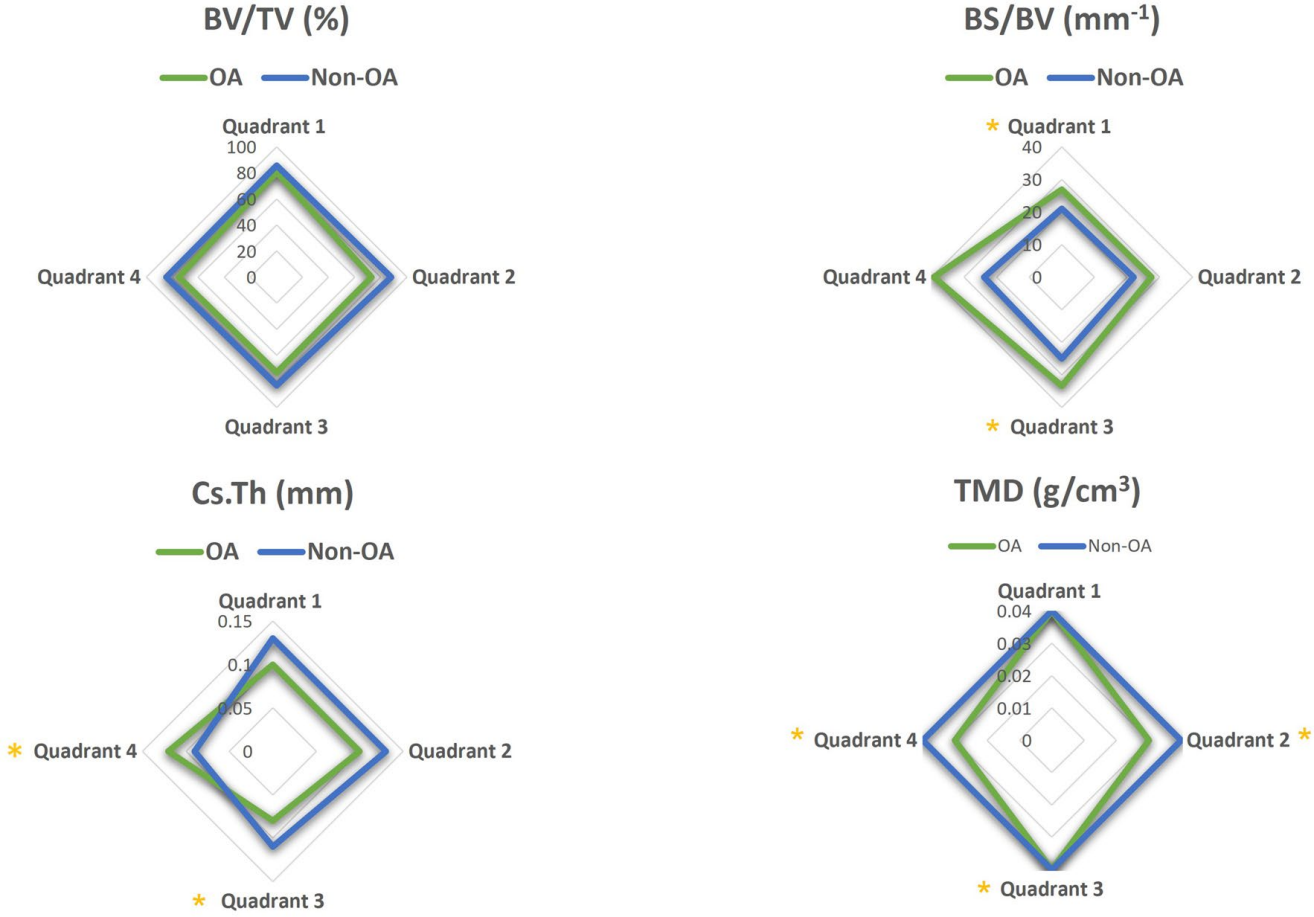
Regional distribution of the median value for the cortical plate microstructural properties in the OA (green) and non-OA (blue) patients. No significant differences were found when cortical plate in different anatomical regions was compared within the OA and non-OA patients. (*) shows when there is a significant difference between OA and non-OA patients (*p* < 0.05). BV/TV: bone volume fraction; BS/BV: specific bone surface; Cs.Th: cortical thickness; TMD: tissue mineral density.

Mapping of subchondral plate architecture across the femoral head quadrants and PCA analysis (Fig. 4B) revealed a significant difference between OA and non-OA patients (*p* = 0.0001); PC1 positively correlated with CLS in all quadrants (Q1—*p* = 0.0019, Q2—*p* = 0.0050, Q3—*p* = 0.0004, Q4—*p* = 0.0359). All cortical measurements contributed to PC1 similarly except for CS.Th which had lower loading. Quadrant-by-quadrant variation in subchondral plate architecture showed significantly greater BS/BV in quadrants 1 (*p* = 0.03) and 3 (*p* = 0.01), and lower TMD in quadrants 2 (*p* = 0.008), 3 (*p* = 0.05) and 4 (*p* = 0.03) in OA compared to non-OA patients (Fig. 7, Supplementary Table S2). Cs.Th in OA patients was significantly lower in quadrant 3 (*p* = 0.02) and higher in quadrant 4 (*p* = 0.01). Examination of quadrant-specific links between these particular plate architectures and CLS (Table 4) showed a significant positive correlation between CLS and both BS/BV and porosity and a negative relationship with BV/TV in quadrants 1 and 3. In addition, Cs.Th in quadrant 3 and TMD in quadrant 2 were also negatively correlated with CLS.

**Table 4:** Correlation between OA severity and microstructural parameters in all quadrants in cortical bone.

Quadrants	BV/TV		BS/BV		Po(tot)		Cs.Th	TMD
	1	3	1	3	1	3	3	2
r	− 0.71	− 0.65	0.56	0.60	0.71	0.65	− 0.85	− 0.62
*p*	0.01	0.02	0.04	0.03	0.01	0.02	0.000	0.02

BV/TV: bone volume fraction; BS/BV: specific bone surface; Po (tot): total porosity; Cs.Th: cortical thickness, TMD: tissue mineral density. The correlation coefficient, r, and *p* values are reported. *p* < 0.01 was considered significant.

### Osteophyte development is regionally-linked with cartilage lesion severity but unrelated to OA changes in the subchondral plate and trabecular compartments

**Figure 8:**
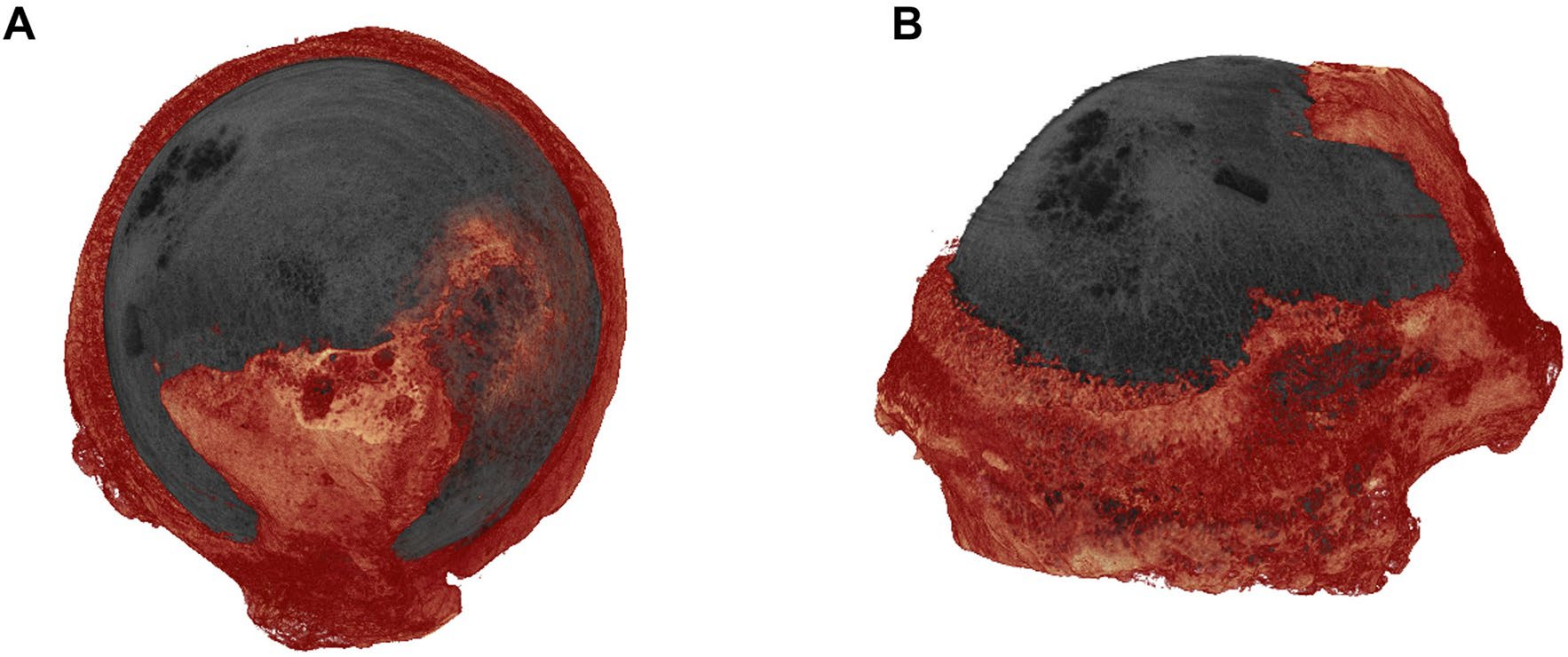
3D representation of OA femoral head epiarticular osteophytes extended from peripheral marginal area to the surface of femoral head; (**A**) axial view and (**B**) coronal view.

Osteophyte is a key characteristic bony feature of OA. Osteophytes were mainly located at the margins of femoral head, margins of fovea and in some cases were extended to the surface of femoral head, forming epiarticular osteophytes (Fig. 8). We, therefore, undertook an evaluation of osteophyte distribution in each quadrant and its spatial relationship with CLS. The evaluation has demonstrated that quadrants 1 and 3 contained osteophytes with greater total volume (TV, Q1: 76.7 mm^3^ [20.5–172.6] and Q3:135.3 mm^3^ [28.3–258.3]) and osteophyte volume (OV, Q1:62.6 mm^3^ [14.9–118.2] and Q3: 75.5 mm^3^ [20.2–202.0]). Moreover, we found that CLS was positively correlated with these osteophyte volumes in quadrants 2, 3 and 4 (Table 5) but there a weak relationship between osteophytes and any OA-related subchondral plate or trabecular compartment changes.

**Table 5:** Correlation between osteophyte formation and OA severity.

Quadrants	TV			OV		
	2	3	4	2	3	4
r	0.81	0.91	0.81	0.81	0.91	0.93
*p*	0.03	0.005	0.02	0.03	0.005	0.003

TV: Total volume of interest; OV: Osteophyte volume. The correlation coefficient, r, and *p* values are reported. *p* < 0.01 was considered significant.

## Discussion

This study investigates the entire femur head to explore whether there are spatial relationships between cartilage lesion severity and structural changes in the local subchondral bone. Previously, such investigations into bone microstructure have been confined only to specific small femoral head segments. The authors postulated that regional analyses of bone microstructure across the entire OA femoral head would reveal new insights into OA pathophysiology, useful to inform earlier OA intervention. We chose to evaluate subchondral plate and trabecular morphometry separately as they may diverge in their contribution or adaptation to OA. The results showed greater articular cartilage deterioration in OA is linked both to a cortical plate with lower BV/TV, TMD and cortical thickness with greater BS/BV and porosity; and to thinner, less separated trabeculae with greater TMD and BS/BV.

Elevated bone remodelling and subchondral bone loss are reported determinants of early OA progression^30^. Results from the Chingford study conducted in a cohort of females (aged 45–64 years) showed that women with progressive OA had greater urinary excretion of NTx and CTx (validated bone resorption markers) than those with non-progressive OA^30^. Consistent with the elevated remodelling/resorption rates which these data suggest, we found that specific bone surface (bone surface area to volume ratio) in both the cortical plate and trabecular compartment was raised in line with CLS scores, thinner trabeculae and a more porous subchondral cortical plate. Increased cortical plate porosity may also reflect the presence of subchondral cysts or cortical breaks. Increased thinning of a more porous subchondral plate has been described in two canine OA models^31^, and thinner trabeculae and reduced plate thickness have also been linked with cartilage damage in a rabbit OA model^32^. Intriguingly, rabbits in which subchondral bone impairment was induced by osteoporosis (OP) prior to surgical OA induction showed aggravated cartilage damage, suggesting that subchondral plate maintenance may protect against OA development. Indeed, it has been reported that thinning of subchondral bone precedes cartilage damage in some animal models^31,33,34^. The results from this study reinforced these findings by demonstrating a clear regionalised thinning of the subchondral bone plate in OA patients which were linked to CLS severity.

Individual differences in femoral head microstructure in OA patients revealed that those with overall low articular surface OA scores, displayed trabecular organisation that resembled non-OA samples (Fig. 5). These patients with low OA scores, in contrast, exhibited clear differences in subchondral plate architecture, indicating that early OA-related modification in the cortical subchondral bone can likely be identified. On this basis, it is tempting to speculate that structural impairment of cortical plate is only later followed by changes in trabecular organisation and that subchondral bone plate thinning may serve as a potential early translatable diagnostic marker for OA.

We found that the mineralisation density of the subchondral plate was lower in OA than non-OA samples, whilst opposing trends were evident in the trabecular compartment. This aligns with previous studies: for example, an examination of specific osteochondral plug regions from human OA tibia plateaus by Cox et al.^35^ showed that bone matrix mineralisation was significantly lower in severe OA (International Cartilage Repair Society grading) and that this reduction in mineralisation was the greatest close to the cartilage, suggesting that cartilage degeneration and bone demineralisation might be linked. Lower mineralisation levels in the subchondral plate in OA femoral heads have also been observed using gravimetric measurements^36^. Grynpas et al.^37^ used density fractionation and chemical analysis and also found that OA results in lowering of bone mineralisation density close to the surface of the subchondral bone of femoral heads, when compared to age-matched and young controls. Diminution of OA subchondral plate mineralisation may also be influenced by bone cysts^18^ as their formation provokes resorption and lower mineralisation. We have also observed a number of subchondral cysts in the examined OA samples and, whilst their potential contribution to the OA progression cannot be ignored, targeting the mechanisms underpinning this reduction in subchondral plate mineralisation density in OA remains an attractive possibility.

There is limited information on the morphometric variation between anatomical femoral head sites and CLS. Although this study demonstrated that there are no statistically significant differences in CLS scores across the four quadrants, it was apparent that quadrants 1 and 3 were markedly affected by OA, with abundant cartilage degeneration and significant differences in the subchondral plate and trabecular microstructure compared to non-OA samples. Quadrants 1 and 3 represent the more weight-bearing posterior surface hip joint regions. The study revealed that with increasing CLS, and hence OA progression, these regions showed the most marked reduction in subchondral plate bone volume fraction, with corresponding increases in porosity and specific bone surface fraction. In trabecular bone, thickness and separation decreased while specific bone surface area and bone mineral density increased in these quadrants. These findings suggest that there is a spatial relationship between weight-bearing, CLS and marked microstructural modifications. These data suggest that early clinical diagnosis of OA should focus upon microstructural examination of the subchondral bone in these weight-bearing regions.

Osteophyte formation is another feature of OA joint pathology. The examination of osteophyte formation revealed that the progressive loss of articular cartilage is accompanied by increased osteophytogenesis. Samples presented marginal and epiarticular osteophytes (Fig. 8). Since higher TMD has been reported in OA patients compared with healthy controls^38–42^, therefore, the relationship between bone mineral density and the presence of osteophytes was determined. However, the examination suggested that osteophyte formation was not related to TMD.

This is the first study that examined the spatial relationship between cartilage degeneration and the remodelling of the underlying subchondral bone. Whilst this study reveals several novel relationships between femoral head bone microstructure and CLS, several caveats need to be exercised in their interpretation. The number of subjects was not large in this study (7 OA and 6 Control specimens) due to restrictions for accessing human explants. A power analysis based on a significance of *p* = 0.05 showed that the minimum number of samples to achieve an overall significance with a power of 0.8 was 17 for each OA score. A large study would be required to validate the findings obtained in this study. In addition, the OA samples included in this study were from late-OA patients undergoing replacement. It cannot, therefore, be known for certain that the bone and cartilage changes in OA samples with low CLS scores are truly representatives of early OA in young and adolescent patients. The main limitation of this study was the age difference between the OA (47.3 ± 17.7 years) and non-OA (79.2 ± 6.1 years) group. Although a study by Li et al.^16^, showed no correlation between age (mean age 69.16 ± 12.33 years, range 37–95 years) and subchondral bone microstructure in human osteoarthritic femoral heads, patients that develop significant OA at a younger age (such as patient 2 and 3) may not have the same bone characteristic of those older patients who typically have a joint replacement. Furthermore, the non-OA samples included were not age matched due to difficulty obtaining human samples. Ageing of the skeleton has been reported to have a remarkable impact on bone homeostasis, and is characterised by deterioration of bone microstructure^43–45^. In normal human femoral heads, the loss of bone quality including a decrease of cortical plate thickness, reduced numbers and thinning of trabeculae, and overall decrease of bone mineralisation is considered to be the most important factor affecting structural integrity with age^46,47^. Nevertheless, the significant subchondral bone changes we found including thinner trabeculae, higher cortical porosity and BS/BV were found in OA patients and not the non-OA individuals. The trabecular TMD was also higher in the OA group which is consistent with previous studies reporting evidence of higher systemic TMD in patients with hip^39,40,48,49^ and spine^50,51^ OA when compared to healthy controls matched for sex and age. Previous interventions/surgical history of OA patients was also not examined, which would have changed possibly the shape of the femoral head, proximal femur + /- acetabulum. A further factor is that the non-OA cohort of patients, those that suffer a hip fracture, typically have osteopenia or osteoporosis. This could bring in a number of other confounding variables.

## Conclusion

In this study, human femoral heads from both OA and non-OA patients were retrieved and the spatial links between subchondral bone architectural features and cartilage degeneration were analysed. We demonstrated that the subchondral plate and trabecular compartments exhibit different characteristics with increasing CLS in OA joints; somewhat expected as they serve differing morphology, physical and mechanical roles. The heterogeneous microstructural changes of subchondral bone that are linked with CLS in different regions of the femoral head could be due to divergence in load distribution in the hip joint and proximal femur. The findings suggest that changes in subchondral plate thickness, porosity, TMD and trabecular bone thickness, TMD and separation could be used as imaging markers for early diagnosis of OA. These data indicate that anatomical characteristics of OA bone microstructure in the femoral head may provide a means for diagnosis of early OA and disease progression in clinics.

## Supplementary Information

Supplementary Information.
