# Supplementary material for: Spatial links between subchondral bone architectural features and cartilage degeneration in osteoarthritic joints
Source: Sci Rep. 2022 Apr 23;12:6694. doi: 10.1038/s41598-022-10600-6 (PMC9035167; doi:10.1038/s41598-022-10600-6)
Supplement: Supplementary file 1 — Supplementary Information. [file 41598_2022_10600_MOESM1_ESM.docx]

**Spatial links between subchondral bone architectural features and cartilage degeneration** **in osteoarthritic joints**

**Sara Ajami*^1, 2^, Behzad Javaheri^3^, Y-M Chang^4^, Nimalan Maruthainar^5^, Tahir Khan^6^, James Donaldson^6^, Andrew A Pitsillides^4^, Chaozong Liu^1^**

1. Institute of Orthopaedics and Musculoskeletal Science, University College London, Royal National Orthopaedic Hospital, Stanmore HA7 4LP, United Kingdom
2. Great Ormond Street Institute of Child Health, University College London, London WC1N 1EH
3. School of Mathematics, Computer Science and Engineering, City University of London, London, UK
4. Comparative Biomedical Sciences, The Royal Veterinary College, Royal College Street, London, NW1 0TU, United Kingdom
5. Department of Orthopaedic Surgery, Royal Free Hospital, London, United Kingdom
6. The Royal National Orthopaedic Hospital, Brockley Hill, Stanmore HA7 4LP, United Kingdom

**Supplementary material**

**Tables**

Table S1: Tissue mineral density (TMD) of the trabecular bone measured in the four different quadrants for OA and non-OA samples

| Group | Patients | Quadrant 1 | Quadrant 2 | Quadrant 3 | Quadrant 4 |
| --- | --- | --- | --- | --- | --- |
| OA | P1 | 0.505 | 0.392 | 0.481 | 0.304 |
|  | P2 | 0.104 | 0.021 | 0.017 | 0.011 |
|  | P3 | 0.465 | 0.420 | 0.408 | 0.371 |
|  | P4 | 0.368 | 0.326 | 0.300 | 0.335 |
|  | P5 | 0.287 | 0.341 | 0.232 | 0.352 |
|  | P6 | 0.386 | 0.373 | 0.341 | 0.388 |
|  | P7 | 0.510 | 0.396 | 0.569 | 0.506 |
| Non-OA | P8 | 0.232 | 0.323 | 0.192 | 0.382 |
|  | P9 | 0.049 | 0.056 | 0.154 | 0.213 |
|  | P10 | 0.057 | 0.008 | 0.140 | 0.074 |
|  | P11 | 0.150 | 0.231 | 0.171 | 0.237 |
|  | P12 | 0.166 | 0.211 | 0.075 | 0.159 |
|  | P13 | 0.077 | 0.201 | 0.103 | 0.054 |

Table S2: Tissue mineral density (TMD) of the cortical bone measured in the four different quadrants for OA and non-OA samples.

| Group | Patients | Quadrant 1 | Quadrant 2 | Quadrant 3 | Quadrant 4 |
| --- | --- | --- | --- | --- | --- |
| OA | P1 | 0.037 | 0.036 | 0.035 | 0.035 |
|  | P2 | 0.031 | 0.032 | 0.029 | 0.036 |
|  | P3 | 0.034 | 0.035 | 0.035 | 0.034 |
|  | P4 | 0.037 | 0.037 | 0.036 | 0.035 |
|  | P5 | 0.037 | 0.037 | 0.036 | 0.036 |
|  | P6 | 0.034 | 0.035 | 0.034 | 0.034 |
|  | P7 | 0.036 | 0.035 | 0.037 | 0.034 |
| Non-OA | P8 | 0.036 | 0.037 | 0.037 | 0.036 |
|  | P9 | 0.036 | 0.036 | 0.037 | 0.036 |
|  | P10 | 0.038 | 0.037 | 0.038 | 0.036 |
|  | P11 | 0.038 | 0.038 | 0.036 | 0.038 |
|  | P12 | 0.037 | 0.037 | 0.035 | 0.035 |
|  | P13 | 0.039 | 0.037 | 0.037 | 0.036 |

.
